# Establishment of an Efficient Polyethylene Glycol (PEG)-Mediated Transformation System in *Pleurotus eryngii* var. *ferulae* Using Comprehensive Optimization and Multiple Endogenous Promoters

**DOI:** 10.3390/jof8020186

**Published:** 2022-02-14

**Authors:** Qi Zhang, Liting Zhao, Mengye Shen, Jingyun Liu, Youran Li, Sha Xu, Lei Chen, Guiyang Shi, Zhongyang Ding

**Affiliations:** 1 Key Laboratory of Carbohydrate Chemistry and Biotechnology, Ministry of Education, School of Biotechnology, Jiangnan University, Wuxi 214122, China; 18811992762@163.com (Q.Z.); 7190201028@stu.jiangnan.edu.cn (L.Z.); smy_jnedu@163.com (M.S.); jingyunliu214@163.com (J.L.); leichen@jiangnan.edu.cn (L.C.); gyshi@jiangnan.edu.cn (G.S.); 2 National Engineering Research Center for Cereal Fermentation and Food Biomanufacturing, Jiangnan University, Wuxi 214122, China; liyouran@jiangnan.edu.cn (Y.L.); xusha1984@jiangnan.edu.cn (S.X.); 3 Jiangsu Provincial Engineering Research Center for Bioactive Product Processing, Jiangnan University, Wuxi 214122, China

**Keywords:** *Pleurotus eryngii* var. *ferulae*, protoplast, genetic transformation, heterologous gene expression, endogenous promoters

## Abstract

*Pleurotus eryngii* var. *ferulae*, a fungus of the genus *Pleurotus*, efficiently degrades lignin, especially during co-cultivation with other fungi. However, low transformation efficiency and heterologous gene expression restrict systematic studies of the molecular mechanisms and metabolic control of natural products in this mushroom. In this study, the homologous resistance marker carboxin (*cbx*) was used to establish a polyethylene glycol-mediated transformation (PMT) system in *P. eryngii* var. *ferulae*. Optimization of the transformation process greatly improved the number of positive transformants. In particular, we optimized: (i) protoplast preparation and regeneration; (ii) screening methods; and (iii) transformation-promoting factors. The optimized transformation efficiency reached 72.7 CFU/μg, which is higher than the average level of *Pleurotus* sp. (10–40 CFU/μg). Moreover, three endogenous promoters (P*_pfgpd1_*, P*_pfgpd2_*, and P*_pfsar1_*) were screened and evaluated for different transcription initiation characteristics. A controllable overexpression system was established using these three promoters that satisfied various heterologous gene expression requirements, such as strong or weak, varied, or stable expression levels. This study lays the foundation for recombinant protein expression in *P. eryngii* var. *ferulae* and provides a method to investigate the underlying molecular mechanisms and secondary metabolic pathway modifications.

## 1. Introduction

*Pleurotus eryngii* var. *ferulae*, a member of the *Pleurotus* species, is a tetrapolar heterothallic edible mushroom [1]. The wild-type strain is mainly distributed in Europe, the Middle East, North Africa, along the Mediterranean coast, and China [2,3]. *P. eryngii* var. *ferulae* is edible and has several medicinal properties, including anti-tumor, antioxidant, anti-obesity, and immunoregulatory properties [4,5,6,7]. Moreover, *P. eryngii* var. *ferulae* secretes large amounts of lignin-degrading enzymes (laccase, manganese peroxidase, and lignin peroxidase) for industrial use [8]. These enzymes can be used to degrade lignin, provide bioenergy, decolor dyes, and treat sewage [9]. Our previous studies confirmed that co-cultivating *P. eryngii* var. *ferulae* with certain fungi can increase the transcription of multiple laccase isoenzymes [10,11]. However, the lack of an efficient genetic transformation system restricts further research on the regulatory mechanisms and prevents metabolic engineering.

Common genetic transformation methods used in mushrooms mainly include the following five methods: *Agrobacterium tumefaciens*-mediated, electroporation, liposome-mediated, restriction enzyme-mediated integration (REMI), and polyethylene glycol-mediated transformation (PMT) methods [12,13]. Compared with the other four methods, PMT has high conversion efficiency, mild reaction conditions, a convenient process, and low cost [12]. Recently, PMT has been successfully established in some filamentous fungi, such as *Ganoderma lucidum*, *Laccaria bicolor*, *Coprinus cinereus*, *Flammulina velutipes*, *Cordyceps militaris*, and *Lentinula edodes* [14,15,16,17,18,19,20]. However, sturdy hyphae, complex cell wall structures, low integration efficiency, and the low expression efficiency of heterologous genes in filamentous fungi limit the application of these fungi for research. Therefore, an efficient genetic transformation system must be developed.

The cell wall of mushroom mycelium is mainly composed of glucan and chitin. These substances form thick, strong regions that block the entry of genetic materials [17,21]. Hence, preparing high-quality protoplasts is the most critical step for genetic transformation. Additions of carrier DNA and functional substances during transformation play an auxiliary role in transformation efficiency. Strong promoters facilitate the high-level transcription and expression of heterologous genes in fungi. Homologous promoters are conducive to RNA polymerase and transcription factor recognition, reduce methylation, and improve transcription initiation efficiency [22]. The transcription initiation efficiencies of the 35S promoter (P*_CaMV35S_*) from the cauliflower mosaic virus, the glyceraldehyde-3-phosphate dehydrogenase promoter (P*_gpd_*), and the small GTPase promoter (P*_ras_*) from *L. edodes* are all far lower than those of homologous promoters in *G. lucidum* [23]. Our previous studies found that *pfgpd1*, *pfgpd2*, *pfsar1* (small COPII coat GTPase gene), and *pfras* all maintain high transcription levels under diverse culture conditions [24]. Intracellular expression of enhanced green fluorescent protein (EGFP) can be used to verify the transcription initiation efficiency of these promoters and further clarify promoter selection for a new strain genetic transformation system.

This study describes a genetic transformation system for *P. eryngii* var. *ferulae.* We improved the transformation efficiency and positive transformant rate by optimizing the three core steps in the transformation process: protoplast preparation and regeneration, PEG-mediated transformation, and the effect of different added substances. Further, *egfp* was used as a reporter gene to compare the transcriptional initiation efficiency of four endogenous promoters in *P. eryngii* var. *ferulae*. This work provides an important foundation for research on regulatory mechanisms and metabolic engineering using *P. eryngii* var. *ferulae*.

## 2. Materials and Methods

### 2.1. Strains and Culture Medium

*P. eryngii* var. *ferulae* JM301 (CCTCC AF 2019009) was obtained from the China Center for Type Culture Collection. The fungi were cultured as follows: preculture medium (0.11 M glucose, 10 g/L peptone, 5 g/L yeast extract, 7.35 mM KH_2_PO_4_, 4.06 mM MgSO_4_·7H_2_O, and 0.33 mM vitamin B1) was used for primary shake-flask cultivation and secondary static cultivation. Potato dextrose broth (PDB) medium comprised 200 g/L potato decoction and 0.11 M glucose. Wheat bran medium (WBM) consisted of 20 g/L wheat bran, 20 g/L corn powder, 0.11 M glucose, and 1 mM K_2_SO_4_ (pH 9.0). CYM medium contained 0.029 M maltose, 0.11 M glucose, 2 g/L yeast extract, 2 g/L tryptone, 4.17 mM MgSO_4_, and 0.34 M KH_2_PO_4_; 0.6 M sucrose was added to CYM medium to generate CYM regeneration medium, which was used to regenerate *P. eryngii* var. *ferulae* protoplasts.

### 2.2. Resistance Gene Selection and Fungal Sensitivity

Antibiotic sensitivity was determined using CYM medium with varying concentrations of hygromycin B (*hygB*, Macklin, Shanghai, China) (0, 50, 100, and 200 mg·L^−1^) or carboxin (*cbx*, Macklin) (0, 1.0, 2.0, and 4.0 mg·L^−1^). Mycelia were cultured at 25 °C for 7 days.

### 2.3. Plasmid Construction

According to the test of fungal sensitivity, *cbx* resistance was used to screen positive transformants. Referring to a previous study [25], *cbx* is a 1,4-oxathiin derivative, which blocks the growth of basidiomycetes by inhibiting the activity of the succinate dehydrogenase B subunit (SDHB). The mutant SDHB with an amino acid substitution (His239 to Leu) confers resistance to *cbx*. *Sdhb* was amplified using genomic DNA as a template using the primers listed in Table S1. The complete *pfsdhb* sequence (1905 bp) was obtained using gene walking technology. This sequence consists of a promoter, the *Sdhb* open reading frame (ORF), and a terminator. His (C**A**C) at position 239 of SDHB was replaced with Leu (C**T**C) via site-directed mutagenesis. The mutant *Pfsdhb* fragment was inserted into the pMD19T simple vector backbone (Takara) to generate the pKAB plasmid (Figure S1).

To enhance intracellular heterologous gene expression, four endogenous promoters were tested for transcription initiation efficiency. Four genes, *pfgpd1* (KDQ24107.1), *pfgpd2* (KDQ24081.1), *pfsar1* (KDQ33428.1), and *pfras* (KDQ24534.1) were amplified using *P. eryngii* var. *ferulae* genomic DNA as a template. The four promoters were obtained using gene walking technology (Table S1). The transcription start site was determined by the Berkeley Drosophila Genome Project (http://www.fruitfly.org/seq_tools/promoter.html, accessed on 12 September 2021) and the distribution of cis-acting response elements on the promoter was determined using PlantCARE (http://bioinformatics.psb.ugent.be/webtools/plantcare/html/, accessed on 12 September 2021). The endogenous promoters and the *pfsdhb* terminator were inserted into the pKAB plasmid to generate the pKAB1–pKAB4 plasmids (Figure S1). There are two restriction sites (*XbaI* and *SalI*) for heterologous gene insertion.

### 2.4. Preparation of Protoplasts

Mycelia were grown in preculture medium and collected by filtration through gauze. Then, the tissue was rinsed three times with 0.6 M mannitol. The mycelia were incubated for 1.0 h in 15 mL of 10 mg/mL lywallzyme (Guangdong Institute of Microbiology, Guangdong, China) in 0.4 M mannitol at 30 °C. Optimized conditions include osmotic pressure stabilizers (mannitol, sorbitol, MgSO_4_, sucrose, or KCl), osmotic pressure stabilizer concentration (0.2, 0.4, 0.6, 0.8, and 1.0 mol/L), osmotic pressure stabilizer (10, 15, 20, 25, and 30 mg/mL), reaction time (1, 2, 3, 4, and 5 h), and reaction temperature (22, 26, 30, 34, and 38 °C). Protoplasts were separated by filtration through a 40 µm cell strainer, collected by centrifugation at 3500 *g* for 10 min at 4 °C, washed twice with 15 mL STC buffer (0.6 M sorbitol, 10 mM CaCl_2_, and 10 mM Tris-HCl pH 7.5), resuspended in STC buffer, and stored at 4 °C until use.

### 2.5. PEG-Mediated Protoplast Transformation

One hundred microliters of STC buffer containing 10^8^ mL^−1^ protoplasts were gently mixed with 10 mg plasmid, functional substances (salmon sperm deoxyribonucleic acid (SS-DNA), lambda DNA (λDNA), spermidine, or heparin), and 150 μL PTC buffer (40% PEG4000, 50 mM CaCl_2_, and 10 mM Tris-HCl pH 7.5). The mixture was incubated on ice for 10 min. Then, 1 mL PTC buffer was added, and the mixture was incubated for an additional 30 min at 28 °C. The protoplasts were recovered by centrifugation (4 °C, 5 min at 4000 *g*) and resuspended in 1 mL CYM regeneration medium. Then, the protoplasts were statically cultured at 25 °C for 0–4 days (post-transformation culture without *cbx* resistance). The transformed protoplasts were incubated in monolayers or double layers in CYM regeneration medium with 2 mg/mL *cbx* for 10–14 days at 25 °C. Monolayer screening denotes that there is only one layer of the resistance medium in the plate. Double-layer screening means that the bottom medium contains no resistance medium and the upper medium contains *cbx* for transformant selection. Each transformant was cultured through five passages in medium containing 4 mg/mL *cbx*.

### 2.6. Transformant Verification

*pfsdhb* was amplified using genomic DNA from the wild-type strain and transformants as templates. The molecular weight of the fragments was determined by agarose gel electrophoresis. The nucleic acid sequence and mutation site were determined by Sangon Biotech Company (Shanghai, China) [26].

### 2.7. Fluorescence Intensity Analysis

*Egfp* was inserted into the multiple cloning site of pKAB1–pKAB4 according to the above method. The transformants were transferred to CYM medium and cultured for 7 days at 25 °C while being shaken at 150 rpm. The hyphae were removed with tweezers and placed on a glass slide. A confocal laser microscope (Leica, Wetzlar, Germany) was used to compare the difference in green fluorescence intensity in the hyphae of different transformants.

### 2.8. RT-qPCR

The four transformants were cultured in PDB and WBM medium for 7 days. A Biospin Plant Total RNA Extraction Kit (BIOER, Hangzhou, China) was used to extract mycelial RNA from all transformants. cDNA was constructed using HiScript III RT SuperMix for qPCR containing gDNA wiper (Vazyme, Nanjing, China), as described previously [27]. The primers were designed by the primer-blast of the NCBI website (https://www.ncbi.nlm.nih.gov/tools/primer-blast/, accessed on 24 November 2021; Table S1). *Sar1* was selected as the internal reference gene [28]. The target genes were quantified using the 2^−ΔΔCt^ method. At least three independent biological and technical replicates were performed for each sample.

### 2.9. Statistical Analysis

Data were presented as the mean ± standard deviation (SD). Duncan’s multiple range tests (*p* ≤ 0.05) were used for data analysis. SPSS v.11.4 (IBM Corp., Armonk, NY, USA) was used to process data.

## 3. Results

### 3.1. Resistance Gene Selection and Fungal Sensitivity

**Figure 1:**
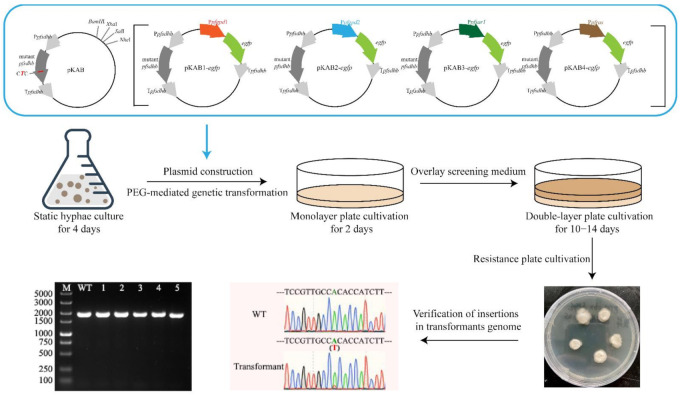
The basic steps of the PEG-mediated protoplast transformation and transformant verification in *P. eryngii* var. *ferulae*. The process includes static hyphae culture, monolayer plate cultivation, double-layer plate cultivation, re-screening, and transformant verification. The *pfsdhb* gene is amplified using the wild-type and transformant genomes as templates. Lane WT, wild-type strain; Lanes 1–5, the strain transformed with pKAB. PCR products amplified from the transformant or wild-type strains were sequenced. The position of the bracket indicates the mutated nucleotide.

Wild-type *P. eryngii* var. *ferulae* was extremely sensitive to *cbx*; 2 mg/L *cbx* completely inhibited mycelial growth (Figure S2a). In contrast, 200 mg/L *hygB* achieved the same effect (Figure S2b), which indicates that selection with *cbx* is sensitive and has low cost. Plasmid pKAB containing the mutant *pfsdhb* gene was transformed into *P. eryngii* var. *ferulae* via PMT (Figure 1). Once pKAB was integrated into the genome, the transfected gene was stably maintained in the genome even after five passages. The *pfsdhb* fragment was amplified and sequenced using the transformant genome as a template. We observed two alleles at the mutation site (Figure 1), indicating that the transformant genome contained both original and mutant *pfsdhb* and that the heterologous gene integration method was insertion rather than replacement. The original *pfsdhb* gene was retained while the mutant *pfsdhb* from pKAB existed independently in the *P. eryngii* var. *ferulae* genome for replication and transcription. A similar phenomenon appeared for this fragment during *G. lucidum* transformation [29]. Thus, a genetic transformation system was established for the first time in *P. eryngii* var. *ferulae*.

### 3.2. Protoplast Preparation and Regeneration of P. eryngii var. ferulae

**Figure 2:**
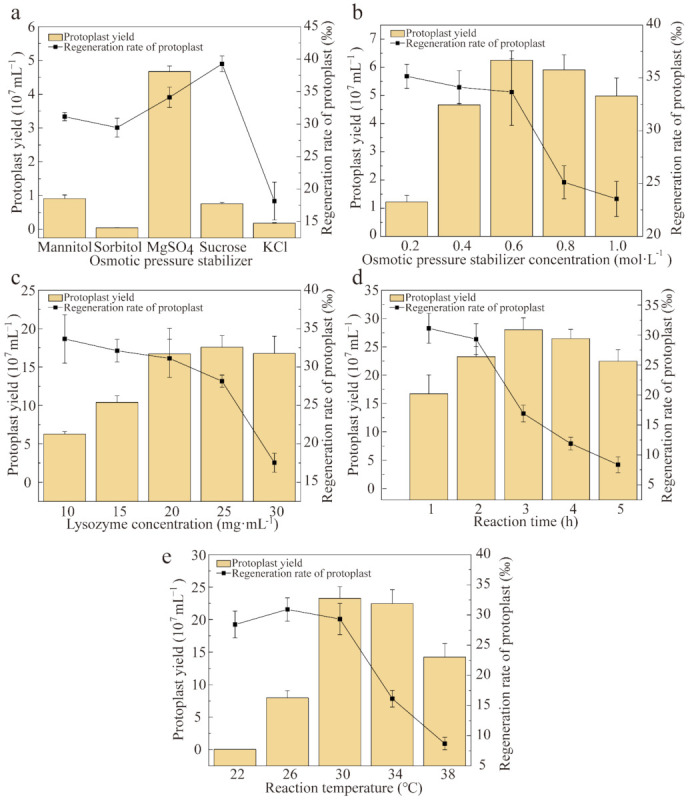
Protoplast preparation and regeneration under different reaction conditions. (**a**) Osmotic pressure stabilizers; (**b**) MgSO_4_ as the osmotic pressure stabilizer; (**c**) lysozyme concentration; (**d**) Rreaction time; and (**e**) reaction temperature. The protoplast yield and regeneration rate were determined for each reaction condition.

Only a few positive *P. eryngii* var. *ferulae* transformants were obtained using the initial transformation conditions. Improving the quantity and quality of *P. eryngii* var. *ferulae* protoplasts seemed the most basic solution. There are noteworthy differences in the reagents and methods used to prepare mushroom protoplasts, even for closely related species. Lysozyme in MgSO_4_ buffer solution generated the largest number of *P. eryngii* var. *ferulae* protoplasts (Figure 2 and Figure S3). Mannitol and sucrose, which are typically used for *P. eryngii* and *P. ostreatus*, respectively, are not suitable for *P. eryngii* var. *ferulae* mycelia [30,31]. One reason is that lysozyme has variable substrate selectivity in different osmotic pressure stabilization buffers. Another reason is the diverse chitin and β-1,3-glucan content in different mycelial cell walls. Excessively high concentrations of stabilizer and lysozyme, reaction time, and reaction temperature all have a significant negative impact on the regeneration capability of protoplasts. Excessive enzymatic hydrolysis makes fungal cell wall repair difficult. The optimal conditions for protoplast preparation and regeneration were determined for *P. eryngii* var. *ferulae* (Figure 2). We selected 0.6 M MgSO_4_ as the osmotic pressure stabilizer and mycelia were enzymatically hydrolyzed with 20 mg/mL lysozyme (final concentration) at 30 °C for 2 h. This returned 2.33 × 10^8^ CFU/mL protoplasts, and the regeneration rate reached 2.93%.

### 3.3. Transformant Screening Methods

**Figure 3:**
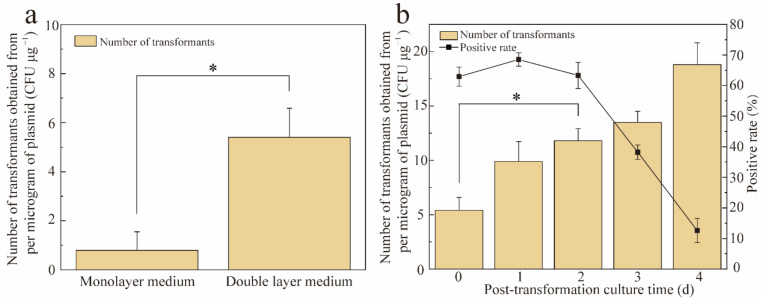
Different screening methods after transformation. (**a**) Culture methods for screening transformants; (**b**) post-transformation culture time. * *p* < 0.05.

Screening methods were also optimized (Figure 3). By adopting a double-layer plate method, the number of transformants obtained greatly improved from 0.8 to 5.4 CFU/μg, which is 6.75 times higher than that obtained using the monolayer plate method. This indicates that when transformants exist in protoplast form, they remain sensitive to *cbx* resistance. Therefore, the resistance-free medium in the lower layer ensures protoplast survival. In addition, increasing the post-transformation culture time improved the transformant yield, but an excessively long post-transformation culture time significantly increased the false positive transformant rate. Therefore, adding a 2-day resistance-free culture step after transformation yielded the highest number of transformants (11.8 CFU/μg) while ensuring a high positive rate.

### 3.4. Effect of Promoting Factors on Enhancing Transformation Efficiency

**Figure 4:**
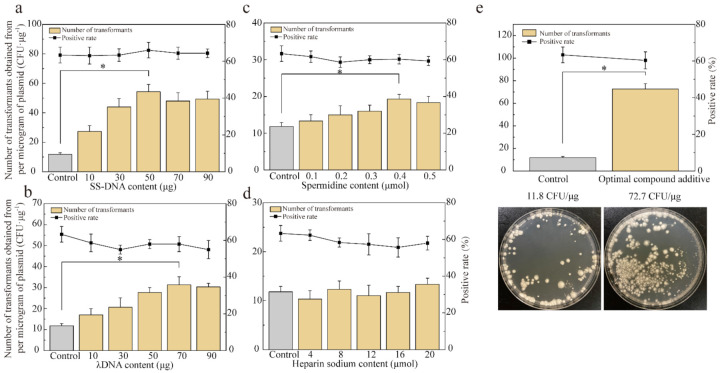
Effect of promoting factors on transformation efficiency. (**a**) SS-DNA; (**b**) λDNA; (**c**) spermidine; and (**d**) heparin sodium. (**e**) Optimal compound additive (50 μg SS-DNA content, 70 μg λDNA, and 0.4 μmol spermidine). Significance was calculated using the highest value in each group and control. * *p* < 0.05.

As the amount of SS-DNA, λDNA, and spermidine increased, the transformation efficiency of the exogenous plasmid in *P. eryngii* var. *ferulae* was higher (Figure 4a–d), with the highest efficiency using 50 μg, 70 μg, and 0.4 μmol, respectively. Heparin sodium had no effect on transformation. Moreover, these four promoting factors did not change the positive transformant rate, which remained at about 60.3%. The optimal additive was composed of 50 μg SS-DNA, 70 μg λDNA, and 0.4 μmol spermidine. This combination achieved the highest transformation efficiency (72.7 CFU/μg), which is 6.16 times higher than that before optimization. This result indicates that these promoting factors do not interfere with each other in the mixed system and that the efficiency of combined use is higher than that of each compound alone.

### 3.5. Effect of Four Endogenous Promoters on EGFP Expression

All transformants (*P. eryngii* var. *ferulae::pfgpd1-egfp*, *P. eryngii* var. *ferulae::pfgpd2-egfp*, *P. eryngii* var. *ferulae::pfsar1-egfp*, and *P. eryngii* var. *ferulae::pfras-egfp*) were cultured using five passages of resistant plates (4 mg/L *cbx*). Fragment 1 (*egfp*) and Fragment 2 (promoter + *egfp*) were amplified using their genomes as templates (Figure S4), which indicated that the heterologous gene expression fragments of the four plasmids were completely inserted into the *P. eryngii* var. *ferulae* genome.

**Figure 5:**
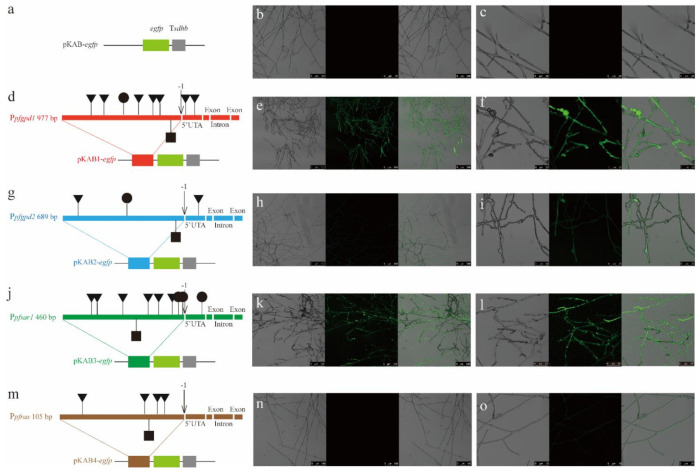
Transcription initiation efficiency of four endogenous promoters in *P. eryngii* var. *ferulae*. (**a**) pKAB-*egfp*, which does not contain any promoter, was used as the negative control; (**d**,**g**,**j**,**m**) P*_pfgpd1_*, P*_pfgpd2_*, P*_pfsar1_*, and P*_pfras_* were analyzed for core cis-responsive elements and inserted into the pKAB-overexpression plasmid to express the *egfp* gene. In the diagrams, filled squares indicate TATA boxes, filled inverted triangles indicate CAAT boxes, and filled circles indicate GC boxes; (**b**,**c**), (**e**,**f**), (**h**,**i**), (**k**,**l**). (**n**,**o**) Phase-contrast, fluorescence, and merged images of transformant mycelium, including *P. eryngii* var. *ferulae*::*pfgpd1*-*egfp*, *P. eryngii* var. *ferulae*::*pfgpd2*-*egfp*, *P. eryngii* var. *ferulae*::*pfsar1*-*egfp*, and *P. eryngii* var. *ferulae*::*pfras*-*egfp*. The scale bars in (**b**,**e**,**h**,**k**,**n**) are 25 µm and the scale bars in (**e**,**f**,**i**,**l**,**o**) are 100 µm.

To ensure heterologous gene expression, the transcription initiation efficiency of the four endogenous promoters was compared using the fluorescence intensity of mycelia (Figure 5). The hyphae of *P. eryngii* var. *ferulae*::*pfgpd1-egfp* and *P. eryngii* var. *ferulae*::*pfsar1-egfp* showed strong fluorescence intensity in each hypha. In contrast, the fluorescence of *P. eryngii* var. *ferulae::pfgpd2-egfp* was weak, while fluorescence from *P. eryngii* var. *ferulae::pfras-egfp* was not observed.

The type and number of core cis-acting elements on the four endogenous promoters (P*_pfgpd1_*, P*_pfgpd2_*, P*_pfsar1_*, and P*_pfras_*) were predicted and analyzed using PlantCARE (Figure 5). All four promoters contain a TATA box, which defines the initial binding position of RNA polymerase II. There was a significant difference in the numbers of CAAT and GC boxes. P*_pfgpd1_* and P*_pfsar1_* had five and six CAAT boxes and one and two GC boxes, respectively. CAAT and GC boxes belong to the transcriptional regulatory region, which is involved in transcriptional activation [32,33]. Therefore, P*_pfgpd1_* and P*_pfsar1_* have strong transcription initiation efficiency. In contrast, we observed only one CAAT box and one GC box in P*_pfgpd2_* and no GC boxes in P*_pfras_*. This is directly related to the low frequency of recruitment of RNA polymerase II in these transformants.

**Figure 6:**
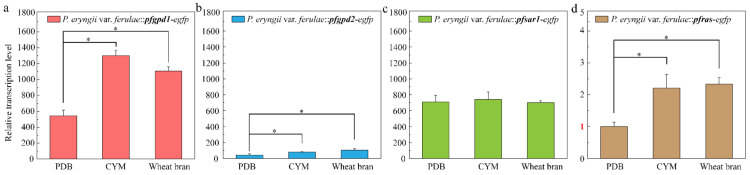
Determination of *egfp* transcription level from four transformants under different culture conditions. The *egfp* gene transcription in *P. eryngii* var. *ferulae*::*pfras*-*egfp* cultured in PDA was used as the control. The relative fold change denotes the ratio of other samples to this sample. (**a**) *P. eryngii* var. *ferulae*::*pfgpd1*-*egfp*; (**b**) *P. eryngii* var. *ferulae*::*pfgpd2*-*egfp*; (**c**) *P. eryngii* var. *ferulae*::*pfsar1*-*egfp*; (**d**) *P. eryngii* var. *ferulae*::*pfras*-*egfp*. * *p* < 0.05.

P*_pfgpd1_* and P*_pfsar1_* had high transcription initiation efficiencies under different culture conditions (Figure 6). P*_pfgpd1_* was especially effective in CYM and WBM culture media. The transcription initiation efficiency of P*_pfsar1_* was consistent using different culture conditions. These results indicate that P*_pfsar1_* is a stable promoter in *P. eryngii* var. *ferulae*; *egfp* transcription driven by P*_pfgpd2_* was relatively low. However, the effect was much better than that of P*_pfras_*. In general, these three important endogenous promoters (P*_pfgpd1_*, P*_pfgpd2_,* and P*_pfsar1_*) exhibit distinct characteristics for expressing heterologous genes and can satisfy a wide range of experimental needs.

## 4. Discussion

Genetic transformation is essential for investigating molecular mechanisms and facilitating mushroom breeding [12]. Previous studies showed that *P. eryngii* var. *ferulae* demonstrates excellent laccase production and dye decolorization capabilities during co-cultivation [11,24,27]. However, the lack of a genetic transformation system is an obstacle that restricts further molecular studies. Transformation methods for mushrooms are complicated and differ greatly between species. For example, the transformation conditions are markedly different for the similar species *P. ostreatus* and *P. eryngii* [34,35]. Therefore, transformation in *P. eryngii* var. *ferulae* must be systematically investigated. In the present study, we optimized *P. eryngii* var. *ferulae* transformation to improve protoplast quantity and quality and to enhance transformation efficiency. We used four endogenous promoters to compare heterologous gene expression in this species.

Mutant *sdhb*, which confers antifungal resistance, results in a 60.3% positive transformant rate. Currently, the most frequently used antibiotic for screening mushrooms is heterologous *hygB* resistance [12]. However, screening with *hygB* has a high false-positive transformation rate. *P. eryngii* var. *ferulae* growth was completely inhibited when treated with 200 mg/L *hygB*, which indicates that the homologous selection marker is better than heterologous resistance. Thus, *cbx* should be the first choice for screening genetic transformation of edible fungi [36].

In this study, the number of positive transformants after comprehensive optimization was approximately 89.9-fold higher than that of the original transformation method. This transformation efficiency was prominent in the entire mushroom transformation system (Table 1). High-quality protoplasts lay the foundation for successful transformation [37]. During the enzymatic hydrolysis of hyphae, lysozyme reduces protoplast viability, especially when using excessive amounts of lysozyme or extended reaction time. Suboptimal reaction conditions increase the number of protoplasts but decrease the vitality, which causes a decreased protoplast regeneration rate.

Optimized transformation conditions determine the number and ratio of positive transformants. Protoplasts are relatively fragile in early growth stages and are more likely to die if they are directly cultured on plates containing antifungal compounds. Adopting the double-layer plate method and increasing the post-transformation culture time provided sufficient recovery time for the transformed protoplasts. Additionally, promoting factors can improve transformation efficiency. Both carrier DNA (SS-DNA and λDNA) reduce plasmid degradation by intracellular nucleases in *P. eryngii* var. *ferulae*. Similarly, adding 50 µg λDNA increases the transformation efficiency of *P. ostreatus* 50-fold [43]. Spermidine can bind to plasmids and initiate endocytosis, which improves plasmid entry into protoplasts [44]. Heparin plays an indispensable role in the genetic transformation of *G. lucidum* [45]. However, 4–20 µmol of heparin sodium had no effect on *P. eryngii* var. *ferulae*. A combination of SS-DNA, λDNA, and spermidine in the transformation reaction increases the *P. eryngii* var. *ferulae* transformation efficiency to reach 72.7 CFU/μg DNA.

Endogenous promoters increase the probability of plasmid integration into the genome and are more easily recognized by the fungus itself to improve transcription initiation [22,23]. This study compared the performance of four endogenous promoters in *P. eryngii* var. *ferulae*. Both P*_pfgpd1_* and P*_pfsar1_* have excellent transcription initiation efficiencies under diverse culture conditions. Multiple CAAT and GC boxes are distributed in the two promoters. In eukaryotes, the number of core cis-acting response elements and the distance from the transcription initiation site play a crucial role in the frequency of transcription initiation [33]. The efficiency of P*_pfsar1_* was not affected by the carbon source, nitrogen source, or polyphenols in the medium. This explains why the *sar1* gene can be used as a housekeeping gene in *P. ostreatus*, owing to its strong promoter stability [28]. Therefore, combined P*_pfsar1_* and P*_pfgpd1_* can replace heterogeneous P*_CaMV35S_* and can be used to construct a dual promoter silencing vector to improve silencing efficiency [46,47]. Only one CAAT box exists in P*_pfgpd2_*, which leads to weak expression. Thus, these three important endogenous promoters (P*_pfsar1_*, P*_pfgpd1_*, and P*_pfgpd2_*) can be utilized to build a comprehensive exogenous protein expression system in *P. eryngii* var. *ferulae*: (i) P*_pfgpd1_* can be used to express and obtain large amounts of exogenous proteins, especially when cultured with CYM and WBM; (ii) stable transcription initiation features of P*_pfsar1_* can be exploited to elucidate molecular mechanisms, such as the mechanism underlying differential regulation of similar transcription factors; and (iii) P*_pfgpd2_* can be used to express cytotoxic proteins, which can reveal the function of target genes while maintaining cell vitality.

## 5. Conclusions

The PMT system was successfully established in *P. eryngii* var. *ferulae* for the first time. High-quality protoplasts laid the foundation for this transformation. Adopting the double-layer plate method, post-transformation culture for 2 days, and the addition of 50 μg SS-DNA, 70 μg λDNA, and 0.4 μmol spermidine during the transformation process maximized transformation efficiency. Moreover, three important endogenous promoters (P*_pfgpd1_*, P*_pfgpd2_*, and P*_pfsar1_*) showed different types of transcription initiation features. A controllable overexpression system can be established using these three promoters, which can be used to select various expression levels. This work provides a method that facilitates studies of molecular mechanisms and novel secondary metabolic pathways in *P. eryngii* var. *ferulae*. This work also proposes a template to establish a genetic transformation system for wild filamentous fungi.

## Figures and Tables

**Table 1:** Summary of protoplast-mediated transformation protocols for different fungal species.

Species	Transformation Methods	Transformation Efficiency (Transformants/μg DNA)	Reference
*P. eryngii* var. *ferulae*	PMT *	72.7	This study
*P. nebrodensis*	PMT	9	[38]
*P. eryngii*	REMI *	10–40	[39]
*P. ostreatus*	PMT	26.7(±11.5)	[40]
*Dichomitus squalens*	PMT	0.8 (±0.3)	[41]
*Grifola frondosa*	PMT	5.6–11.2	[22]
*L. edodes*	REMI	3.6	[36]
*Wolfiporia cocos*	PMT	3	[42]
*G. lucidum*	PMT	15–20	[29]

* PMT: polyethylene glycol-mediated transformation; REMI: restriction enzyme-mediated integration.

## Data Availability

Not applicable. All data is contained within the article.

## Supplementary Materials

The following supporting information can be downloaded at: https://www.mdpi.com/article/10.3390/jof8020186/s1, Figure S1: Construction of the overexpression plasmid, Figure S2: The sensitivity of wild-type *P. eryngii* var. *ferulae* in CYM medium with two different antifungal compounds, Figure S3: Effect of different osmotic pressure stabilizers on protoplast preparation, Figure S4: Verification of the insertion of exogenous plasmids into the four transformant genomes (*P. eryngii* var. *ferulae*::*pfgpd1*-*egfp*, *P. eryngii* var. *ferulae*::*pfgpd1*-*egfp*, *P. eryngii* var. *ferulae*:*pfgpd1*-*egfp*, and *P. eryngii* var. *ferulae*::*pfsar1*-*egfp*), Table S1: qPCR, genome walking, and PCR primers.
