# Supplementary material for: Establishment of an Efficient Polyethylene Glycol (PEG)-Mediated Transformation System in Pleurotus eryngii var. ferulae Using Comprehensive Optimization and Multiple Endogenous Promoters
Source: J Fungi (Basel). 2022 Feb 14;8(2):186. doi: 10.3390/jof8020186 (PMC8876744; doi:10.3390/jof8020186)
Supplement: Supplementary file 1 [file jof-08-00186-s001.zip › jof-1588526-supplementary.pdf]

Supplementary Materials

Captions for supplemental Table and figures

Table S1. qPCR, genome walking, and PCR primers.

Figure S1. Construction of the overexpression plasmid.

Figure S2. The sensitivity of wild-type *P. eryngii var. ferulae* in CYM medium with two different antifungal compounds (**a**) *cbx* resistance; (**b**) *hygB* resistance.

Figure S3. Effect of different osmotic pressure stabilizers on protoplast preparation. The eyepiece and objective of the optical microscope (starMICRO, WuXi, China) were magnified at 10x and 40x, respectively (a) mannitol; (b) sorbitol; (c) $MgSO_4$; (d) Sucrose; (e) KCl.

Figure S4. Verification of the insertion of exogenous plasmids into the four transformant genomes (*P. eryngii* var. *ferulae*::*pfgpd1-egfp*, *P. eryngii* var. *ferulae*::*pfgpd1-egfp*, *P. eryngii* var. *ferulae*::*pfgpd1-egfp*, and *P. eryngii* var. *ferulae*::*pfsar1-egfp*) (**a**) Amplify Fragment1 (*egfp*, 717 bp) in transformants; (**b**) Amplify Fragment2 (promoter + *egfp*) in transformants, which are 1821, 1527, 1324, and 923 bp, respectively. M: marker. NC: negative control.

Supplemental Table

Table S1 qPCR, genome walking, and PCR primers

qPCR primes

Gene	ID	Prime name	Primer sequence (5′—3′)	Amplicon length (bp)
egfp		Fw	TACGTGCAGGAGAGGACCAT	148
		Rv	ACTTGTGGCCGAGGATGTTT	
sar1 [a]	1052294	Fw	GGATAGTCTTCCTCGTCGATAG	133
		Rv	GGGTGCGTCAATCTTGTTAC	

Genome walking primes

Gene	ID	Prime name	Primer sequence (5′—3′)
pfsdhb	KDQ23485.1	S-SP1	TCCAGAAAGAATTCAAGATCTACCG
		S-SP2	CTCGATCCGCGCTTTCTCCACCTCG
		S-SP3	AACGTCTGCGCCCGACAGAACTATC
pfgpd1	KDQ24107.1	G1-SP1	TGGGCCTTGGAAATAAACAA
		G1-SP2	GACTTCGAGCGCGTTACGGA
		G1-SP3	CCAGATGTATTTCGAGTTAG
pfgpd2	KDQ24081.1	G2-SP1	CACCATATAATCGAGATCAA
		G2-SP2	AGGGCGTTACGAAAGACGAT
		G2-SP3	TCCCACTTCCTGATGGACGC
pfsar1	KDQ33428.1	SA-SP1	ACTTGACGTTTCCGATGGCA
		SA-SP2	ATCATTTTTCAACATATGAA
		SA-SP3	TGGCGTTCTTGTGGAGCAGG
pfras	KDQ24534.1	R-SP1	TGATGATAGATGGTCGTATG
		R-SP2	CACCCATGGTGAAAATAGAG
		R-SP3	GGTCAAGGCTGATTTTCCGA

PCR primes

Gene	ID	Prime name	Primer sequence (5′–3′)
Mutant-*pfsdhb*	KDQ23485.1	MS1-Fw	CTAGCTAGCTAGATCGAAGTTATTAGGATCCAGCATAT GTTTTGGGCTGGA
		MS1-Rv	GACGTACAATTGAAGATGGTGAGGCAACGGAACAGA CTCA
		MS2-Fw	TGAGTCTGTTCCGTTGCCTCACCATCTTCAATTGTACGT C
		MS3-Rv	ATTGCCCGAGCCGTTAGATGTCTCA
P_{pfgpd1}		Fw	GCCCAAAACATATGCTGGATCCAGCTTTCTCGCTTCGT CAGAGG
		Rv	GTCGACTAATTTGAGCTCGATTCTAGAGACGTTGACCT ATAAACAAG
P_{pfgpd2}		Fw	GCCCAAAACATATGCTGGATCCCGGCTGTATGGCTTG AGGC
		Rv	GTCGACTAATTTGAGCTCGATTCTAGAGACGTTGACCT

		GCGTACGGGT
P_{pfsar1}	Fw	GCCCAAAACATATGCTGGATCCCGCGAGTGGATCTGTAAGGT
	Rv	GTCGACTAATTTGAGCTCGATTCTAGAGCAGGCCTGCAGACCGAATA
P_{pfras}	Fw	GCCCAAAACATATGCTGGATCCTGCAATCAACGAGCAACAGC
	Rv	GTCGACTAATTTGAGCTCGATTCTAGAGGTGGCCTATATGGCCATCT
T_{pfsdhb}	Fw	TCGAGCTCAAATTAGTCGACACCCTAGTCAACAGCCACGG
	Rv	CGTTCGACGATCTAGCTAGCATTGCCCGAGCCGTTAGATG
egfp	Fw	CGTCTCTAGAATGGGTAAGGGAGAAGAACT
	Rv	GGTGTCGACTTATTTGTATAGTTCATCCA

Supplemental figures

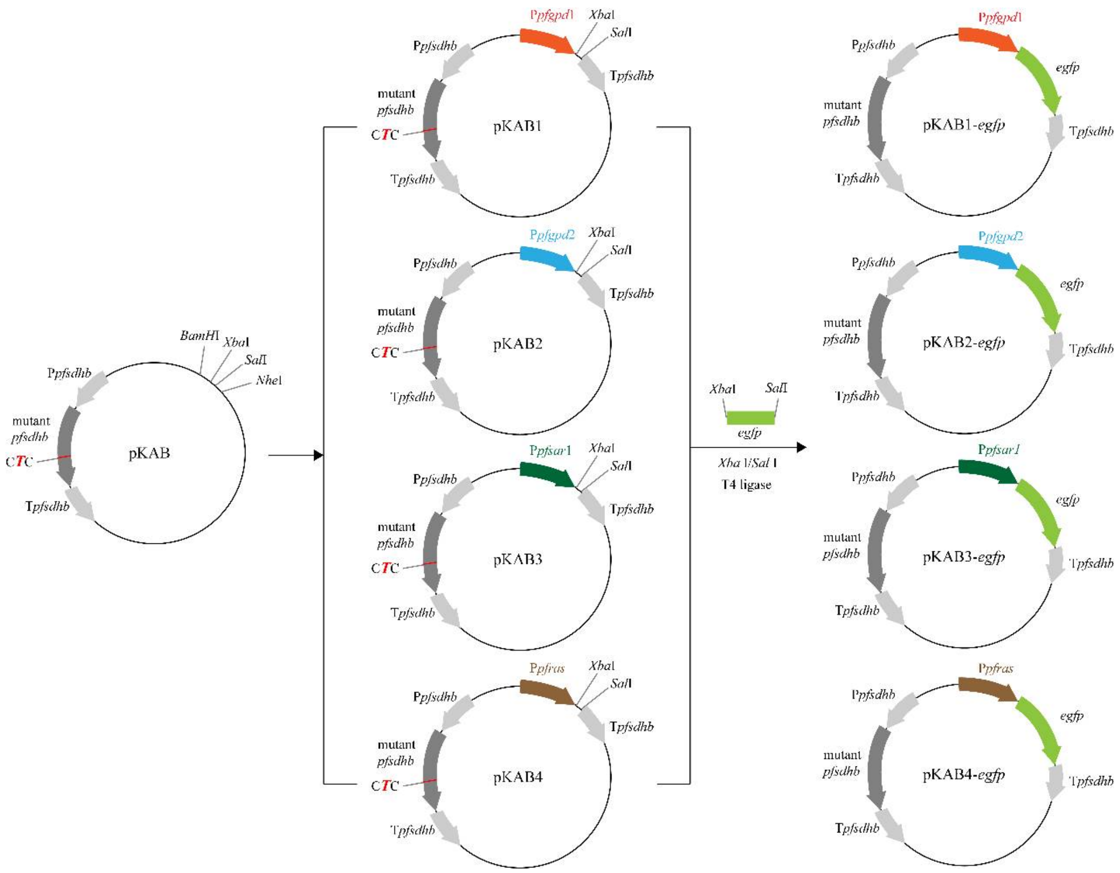

Figure S1. Construction of the overexpression plasmid.

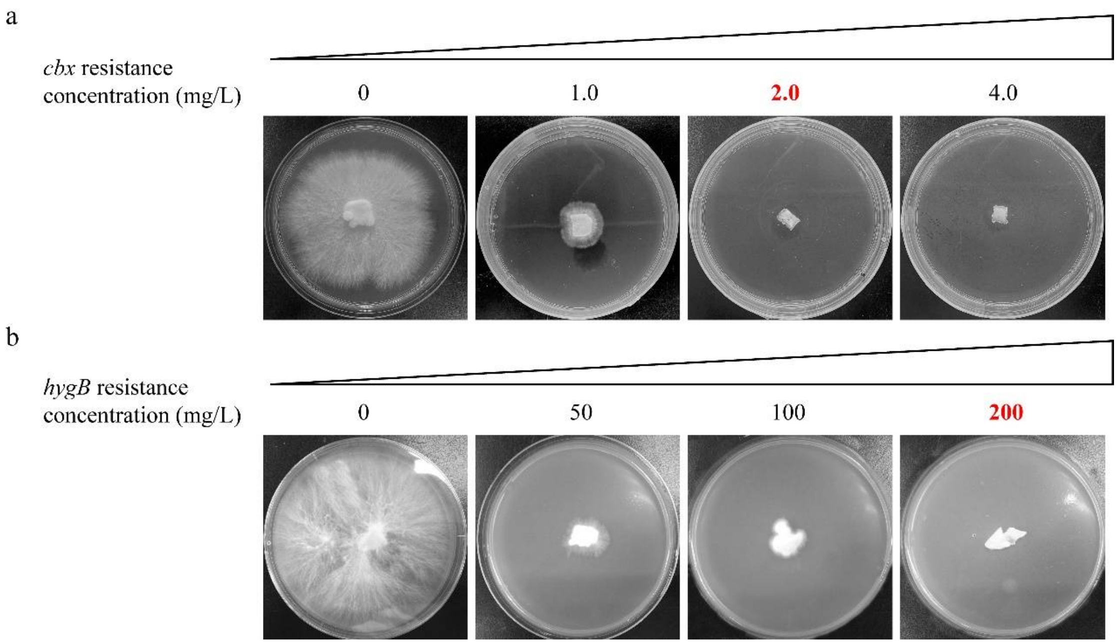

Figure S2. The sensitivity of wild-type *P. eryngii var. ferulae* in CYM medium with two different antifungal compounds (**a**) *cbx* resistance; (**b**) *hygB* resistance.

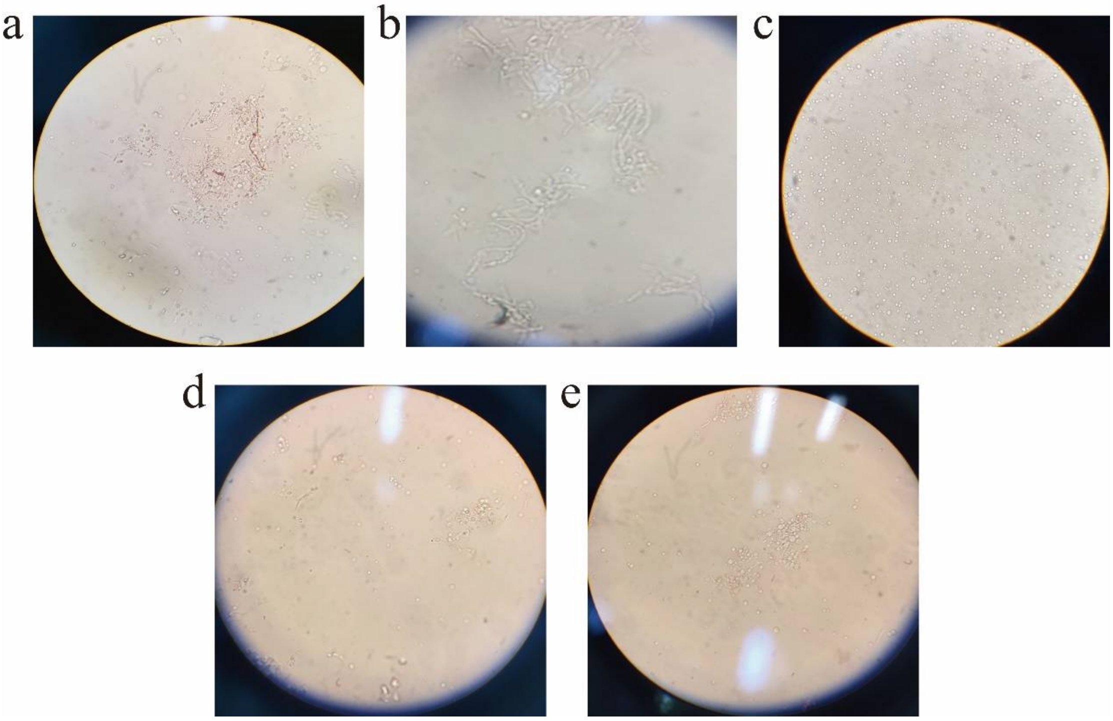

Figure S3. Effect of different osmotic pressure stabilizers on protoplast preparation. The eyepiece and objective of the optical microscope (starMICRO, WuXi, China) were magnified at 10x and 40x, respectively (a) mannitol; (b) sorbitol; (c) $MgSO_4$; (d) Sucrose; (e) KCl.

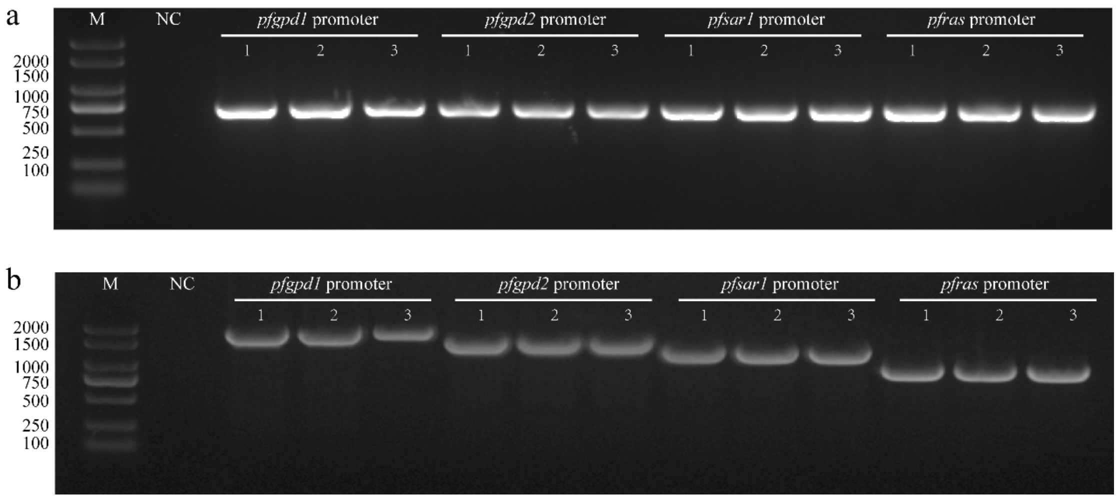

Figure S4. Verification of the insertion of exogenous plasmids into the four transformant genomes (*P. eryngii* var. *ferulae*::*pfgpd1-egfp*, *P. eryngii* var. *ferulae*::*pfgpd1-egfp*, *P. eryngii* var. *ferulae*::*pfgpd1-egfp*, and *P. eryngii* var. *ferulae*::*pfsar1-egfp*) (**a**) Amplify Fragment1 (*egfp*, 717 bp) in transformants; (**b**) Amplify Fragment2 (promoter + *egfp*) in transformants, which are 1821, 1527, 1324, and 923 bp, respectively. M: marker. NC: negative control.
